# Enhancing Procedure Documentation in Neonatal Intensive Care Unit (NICU): A Quality Improvement Initiative at a Tertiary Neonatal Hospital

**DOI:** 10.7759/cureus.60651

**Published:** 2024-05-20

**Authors:** Abrar Al Zaabi, Laila Obaid, Amrat Kumar

**Affiliations:** 1 Neonatology Pediatrics, Tawam Hospital, Al Ain, ARE; 2 Neonatology, Corniche Hospital, Abu Dhabi, ARE

**Keywords:** clinical documentation audit, neonatal care standards, emr compliance training, quality improvement pediatrics, nicu procedure documentation

## Abstract

Background

Accurate and comprehensive procedure documentation in Electronic Medical Records (EMR) is crucial for high-quality patient care, especially in high-acuity settings like Neonatal Intensive Care Units (NICU). Gaps in documentation at Corniche Hospital's NICU that were affecting patient safety and continuity of care were identified and addressed by following a pre and post-intervention design in the research. The process involved the initial audit, educational sessions with healthcare providers, and follow-up audits to measure improvements. Results post-intervention showed a significant increase in compliance with documentation standards, pointing out the effectiveness of educational interventions in improving EMR documentation practices. The local problem is demonstrated through the observation of incomplete and inconsistent procedure documentation in the NICU, hindering effective patient management and multi-disciplinary team communication.

Methods

A Quality Improvement Project (QIP) was implemented, including a baseline audit, educational interventions targeting healthcare providers, and subsequent re-audits to assess improvement. The project involved tailored educational sessions focused on correct EMR usage, adherence to documentation standards, and practical aspects of documenting procedures.

Results

Post-intervention, there was a significant increase in documentation compliance. The percentage of compliance in procedure encounter placement in EMR increased from 81% to 100%, and nursing documentation compliance improved from 11 (52.4%) to 18 (85.7%). However, a slight decrease in the completeness of physician documentation was noted.

Conclusions

The QIP effectively improved procedure documentation in the NICU. Continuous education and periodic review are essential for maintaining and further enhancing documentation standards. This initiative underscores the importance of targeted training and consistent audits in improving clinical documentation in healthcare settings.

## Introduction

Problem description

In the Neonatal Intensive Care Unit (NICU) at Corniche Hospital, Abu Dhabi, a significant challenge was identified in the process of documenting procedures in the Electronic Medical Record (EMR) system. Despite the critical nature of these procedures, it was frequently observed that EMR documentation was either incomplete, inaccurate, or inconsistent. This inadequacy posed a substantial risk to patient safety and continuity of care, especially in a setting where patients, typically preterm neonates or infants with complex conditions, require meticulous and ongoing medical attention. Additionally, incomplete records impeded the efficiency of multi-disciplinary team reviews and follow-up care, often leaving healthcare providers without crucial patient history at critical decision-making junctures.

Available knowledge

The significance of comprehensive and accurate documentation in healthcare, particularly in high-stress and high-acuity environments like NICUs, is well-documented in the literature. Studies have shown that proper documentation directly impacts the quality of patient care and is essential for effective communication among healthcare providers [1]. Furthermore, medical records serve as legal documents and are crucial for audit trails, research, and quality improvement initiatives [2]. However, there exists a notable gap in the literature specifically addressing procedure documentation within NICUs, which this project aims to bridge.

Rationale

The rationale for this Quality Improvement Project (QIP) was rooted in both theoretical and practical considerations. From a theoretical standpoint, the project drew upon principles of clinical documentation improvement (CDI), which emphasize the accuracy, completeness, and timeliness of medical records as a cornerstone of quality patient care [3]. Practically, the project was necessitated by the observed deficiencies in procedure documentation, which not only compromised patient care but also hindered the hospital's adherence to standards set by the Joint Commission International (JCI). The intervention was designed based on the assumption that enhanced training and awareness would improve compliance with documentation standards. This assumption was supported by findings from previous studies indicating that targeted educational interventions can effectively address gaps in healthcare practices [4].

Specific aims

The primary aim of this project was to achieve a 100% compliance rate in procedure documentation within the NICU over a 12-month period. This goal was to be accomplished through a series of educational interventions aimed at healthcare providers, addressing identified gaps in knowledge, experience, and awareness regarding EMR documentation. The project also sought to contribute to the existing body of knowledge on documentation practices in critical care settings, particularly in neonatal care, by providing data-driven insights and practical recommendations for improvement. Additionally, the report aimed to offer a replicable model for similar QIPs in other healthcare settings facing challenges with EMR documentation.

## Materials and methods

Context

The Quality Improvement Project (QIP) was conducted in the Neonatal Intensive Care Unit (NICU) of Corniche Hospital, a tertiary care facility in Abu Dhabi. The NICU is a high-volume, high-acuity unit with a diverse patient population, ranging from preterm to full-term neonates with various medical and surgical conditions. The unit is staffed by a multidisciplinary team of approximately 40 physicians and 150 nurses. The context of a busy, shift-based, and multi-provider environment was a crucial consideration in designing the intervention, as it directly influenced the challenges faced in procedure documentation.

Intervention

The intervention consisted of a series of educational sessions aimed at improving procedure documentation practices. These sessions were designed to address identified gaps in knowledge and skills, focusing on the correct use of the EMR system, adherence to hospital and JCI documentation standards, and practical aspects of documenting procedures accurately and completely. It was carried out by a team comprising NICU physicians, nurses, health information management (HIM) professionals, and clinical educators. This multidisciplinary approach ensured that the intervention addressed the diverse needs and perspectives of the entire NICU team. Table 1 below outlines the key challenges identified in NICU documentation.

**Table 1:** NICU documentation challenges NICU: Neonatal intensive care unit, EMR: Electronic medical records.

Category	Issues
Procedure & knowledge gaps	Not a well-defined procedure
	Multiple steps
	Lack of knowledge
	Unawareness of policy
	Unawareness of EMR
Time & process challenges	Time constraints
	Vague processes
Documentation errors	missing information
	Incomplete procedure
	Incorrect location
	Time difference between team members' entries
	Poorly written

Study of the intervention

The impact of the intervention was assessed using a pre-and post-intervention audit of procedure documentation. The audits evaluated compliance with established documentation standards and accuracy in recording procedures in a total of 21 EMRs reviewed. To ascertain whether observed improvements were attributable to the intervention, the study compared documentation practices before and after the educational sessions. Furthermore, feedback from staff regarding the utility and impact of the training was gathered to supplement the audit findings.

Measures

The primary measure included the rate of compliance with standards for completeness, accuracy, and timeliness of documentation of procedures. Completeness measures the elements present in the documentation, like the process of identification of the patient, the description of the procedure, and the date and time of signature of the provider. Accuracy measured the concordance of procedures documented to procedures done, without errors. Timeliness measures the completion of the documentation within a specific time frame after the performance of a procedure. Secondary measures included staff feedback on the effectiveness of the intervention and changes in documentation practices. The regular meetings within the NICU team could give insight into the challenges and successes. A detailed review of the EMRs was done to establish if the entries met the defined standards. Random checks validated the data collected during the audits.

Analysis

Methodology

Quantitative analysis involved comparing pre- and post-intervention compliance rates using statistical methods, including the Mann-Whitney test for significance. Qualitative analysis was conducted on staff feedback to understand perceptions and attitudes towards the intervention.

Understanding Variation

The analysis included an examination of variation over time, considering factors such as changes in staff, patient demographics, and unit workload.

Ethical considerations

The project was conducted with strict adherence to ethical guidelines, including confidentiality and data protection. No patient-identifiable information was used.

## Results

Initial steps and evolution of the intervention

The QIP began with a baseline audit conducted in April 2020, revealing deficiencies in procedure documentation. Following this, educational sessions were conducted in September 2020. The first re-audit was done between October 2020 and January 2021, with subsequent teaching sessions in April and June 2021. The final re-audit occurred in July to October 2021.

Process measures and outcomes

The initial audit showed only two (9.5%) of procedures were fully documented. Post-intervention, this number increased significantly. Compliance in the placement of a “Procedure encounter” in the EMR rose from 17 (81%) to 21 (100%). Nursing documentation compliance improved from 11 (52.4%) to 18 (85.7%). In addition, physician documentation for including the procedure history in discharge summaries increased from nine (43%) to 18 (85.7%) compliance. However, a slight decline was observed in the completeness of physician documentation, dropping from 21 (100%) to 20 (95%).

Contextual elements interacting with the intervention

The busy and high-stress environment of the NICU, alongside the diverse patient needs, influenced the intervention's implementation and outcomes. Time constraints and the high turnover of staff presented ongoing challenges.

Associations between outcomes and interventions

Significant improvements in documentation were closely associated with the timing of educational interventions. The increased compliance rates post-intervention indicate a direct positive impact of the training sessions.

Unintended consequences and missing data

**Figure 1:**
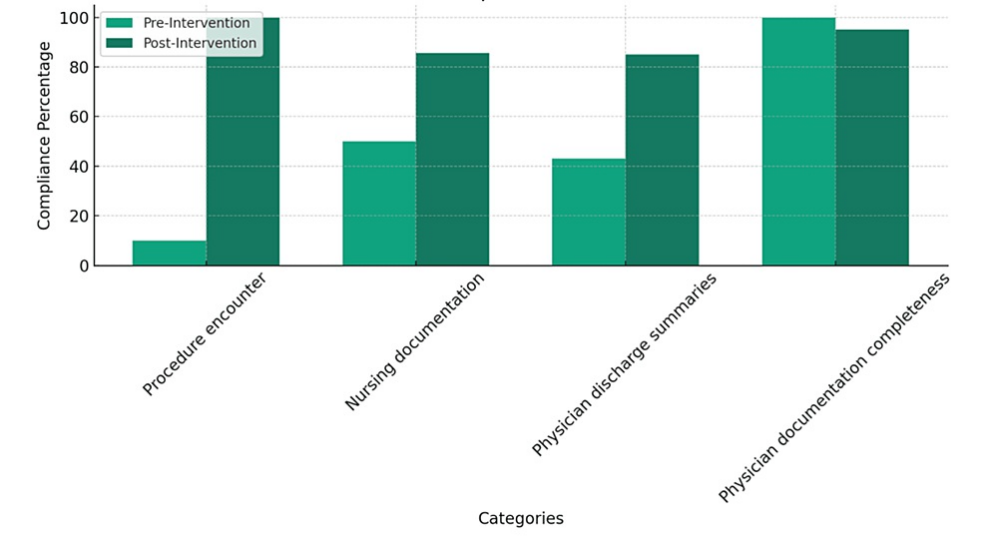
NICU documentation compliance (pre- and post-intervention) The data are represented as percentages (%) of compliance for each category.

Unexpected benefits included enhanced team communication and a more streamlined process for procedure documentation. Challenges included maintaining consistency in documentation practices amidst staff turnover and the high-pressure NICU environment. However, no significant missing data was reported. The audits were comprehensive, covering a representative sample of patient records to ensure a reliable evaluation of the intervention’s impact. The improvements in documentation compliance across various categories within the NICU, before and after the implementation of the intervention, are illustrated in Figure 1 as a percentage.

**Figure 2:**
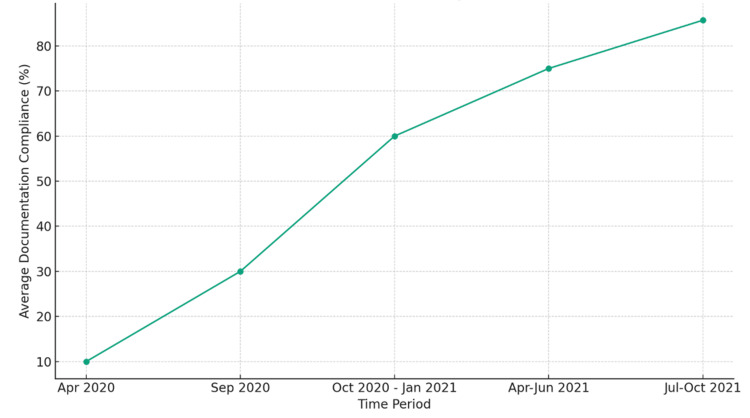
Evolution of documentation compliance over time The data points represent the average percentage of documentation compliance (%) at various time intervals following the educational interventions in the NICU.

The line graph in Figure 2 depicts the progressive increase in average documentation compliance over several time periods following educational interventions in the NICU.

## Discussion

Summary

The QIP demonstrated significant improvement in procedure documentation compliance in the NICU at Corniche Hospital. The compliance rate in procedure encounter placement increased notably from 17(81%) to 21(100%), and nursing documentation compliance improved from 11 (52.4%) to 18 (85.7%). These outcomes align well with the project's rationale and specific aims to achieve 100% compliance in procedure documentation. The project's primary strength was its practical approach, targeting the specific needs of the NICU staff through tailored educational interventions. The involvement of a multidisciplinary team added depth and comprehensiveness to the project's execution.

Interpretation

Association Between Interventions and Outcomes

The positive correlation between the educational interventions and increased documentation compliance suggests that targeted training is effective in improving EMR documentation practices in high-acuity healthcare settings. Although, the differences in outcomes is seen through a slight decline in certain aspects of physician documentation might be attributed to the high-pressure, time-sensitive nature of the NICU environment, highlighting the need for ongoing support and training.

Comparison With Other Publications

Similar improvements have been noted in other studies focusing on EMR documentation, reinforcing the importance of education and training in enhancing clinical documentation [2]. Another study enhanced radiograph quality and reduced repeat radiographs in a NICU by implementing structured educational interventions [5].

Impact on People and Systems

The project not only improved documentation practices but also potentially enhanced patient care and inter-professional communication within the NICU.

Costs and Trade-Offs

While the project required time and resources, the trade-off was a marked improvement in documentation standards, which is crucial for patient safety and care quality.

Improving Documentation in high-acuity settings through electronic health records

A study by Adler-Milstein et al. [6] explores the impact of electronic health records (EHRs) on improving documentation practices in healthcare settings. Their research demonstrates that well-implemented EHR systems enhance the quality of medical documentation, particularly in high-acuity settings like intensive care units, by making the documentation process more efficient and accurate, thus facilitating better patient care and ensuring compliance with healthcare standards.

Standardized documentation templates and education

Implementing standardized documentation templates combined with regular educational sessions on correct Electronic Medical Record (EMR) usage significantly enhances both compliance and the quality of documentation. A study conducted by Goswami et al. [7] in a neonatal neurocritical care unit demonstrated that these interventions increased documentation compliance from 72% to 89% and improved the quality of documentation from 10% to 61% [7].

Continuous quality improvement initiatives

Continuous quality improvement initiatives that encompass regular auditing, feedback, and training are crucial for maintaining improvements in documentation. Root et al. [8] found that auditing neonatal resuscitation procedures and providing feedback based on video and physiological recordings led to increased guideline compliance and improved documentation accuracy [8].

Limitations

The project's findings, while specific to one NICU, highlight issues of generalizability and may not be directly applicable to other settings without considering contextual differences. Internal validity may also have been influenced by factors such as staff turnover and varying levels of experience among NICU staff; however, efforts were made to minimize these through consistent training and re-audits. To further address these limitations, regular feedback sessions and adjustments in training materials were employed.

Challenges and adaptations

Adapting to the complexities of a high-acuity setting such as the NICU involves addressing specific challenges, including varying levels of staff expertise and the high-stress nature of the work. Saravi et al. [9] highlight similar challenges, noting that such environments require tailored approaches to documentation practices to ensure accuracy and completeness, aligning with our findings from the quality improvement project.

## Conclusions

The project underscores the importance of continuous education in enhancing EMR documentation within NICU settings, advocating for ongoing training and periodic audits to sustain improvements in documentation practices. It suggests that the project model could be adapted and implemented in other healthcare environments that face similar challenges. The study emphasizes the need for regular documentation training, particularly in high-acuity healthcare settings, and paves the way for further research in this domain. Future studies are encouraged to examine the long-term impacts of such interventions and consider methods to integrate these practices into regular staff training programs.
